# The Dynamiker cryptococcal antigen lateral flow assay is an accurate, rapid and cost effective test comparable to culture for detection of cryptococcal meningitis

**DOI:** 10.1099/jmm.0.002173

**Published:** 2026-06-25

**Authors:** Maddy Nagwovuma, Richard Kwizera, David Bisagaya Meya, Beatrice Achan

**Affiliations:** 1 Department of Medical Microbiology, School of Biomedical Sciences, College of Health Sciences, Makerere University, Kampala, Uganda; 2 Infectious Diseases Institute, College of Health Sciences, Makerere University, Kampala, Uganda

**Keywords:** cryptococcal meningitis, Dynamiker cryptococcal antigen lateral flow assay (CrAg LFA), diagnostic accuracy, human immunodeficiency virus (HIV), Uganda

## Abstract

**Introduction:** Cryptococcal meningitis (CM) is a leading cause of mortality among people living with human immunodeficiency virus (HIV) in Sub-Saharan Africa, requiring rapid diagnosis. Although the Food and Drug Administration (FDA)-approved Dynamiker cryptococcal antigen lateral flow assay (CrAg LFA) offers a potential point-of-care solution, its use remains limited in high-burden settings.

**Hypothesis/Gap statement:** Despite FDA approval of Dynamiker CrAg LFA for the diagnosis of CM, the underutilization of the test in settings with a high burden of CM is of particular concern. Therefore, there is a need to validate its performance for the diagnosis of CM.

**Aim:** To evaluate the diagnostic performance, turnaround time and cost-effectiveness of the Dynamiker CrAg LFA for CM, using cerebrospinal fluid (CSF) culture as the reference standard.

**Methodology:** Stored CSF samples from 200 HIV-positive patients with suspected or confirmed CM (*n*=245) were tested using the Dynamiker CrAg LFA. Diagnostic metrics were calculated and compared to CSF fungal culture. Time to result and average test cost were also assessed.

**Results:** The Dynamiker CrAg LFA showed a sensitivity of 100% and specificity of 84.9%, with strong agreement (κ=0.83) with CSF culture. Median time to result was 30 s compared to 3 days for culture. The average cost per test was USD 3.41 versus USD 19.80–22.40 for CSF culture.

**Conclusion:** The Dynamiker CrAg LFA is a rapid, accurate and affordable diagnostic tool for CM and holds promise for broader implementation in resource-limited settings.

**Impact Statement**

Cryptococcal meningitis (CM) remains a leading cause of mortality among people living with human immunodeficiency virus (PLHIV) in Sub-Saharan Africa, yet timely diagnosis is hampered by limited access to effective diagnostics. This study evaluated the diagnostic performance of the Dynamiker cryptococcal antigen lateral flow assay (CrAg LFA) in Uganda using cerebrospinal fluid culture as the gold standard. The findings confirm high accuracy, rapid turnaround and a relatively low cost of the Dynamiker CrAg LFA, highlighting its potential to expand diagnostic capacity and improve outcomes in resource-limited settings. These results can inform national policy on point-of-care CM screening and support equitable diagnostic access across healthcare levels.

## Data summary

The authors confirm all supporting data, code and protocols have been provided within the article or through supplementary data files, available with the online version of this article.

## Introduction

Cryptococcal meningitis (CM) is an invasive opportunistic fungal infection of the central nervous system that affects 194,000 new individuals per year globally [1]. In Sub-Saharan Africa (SSA), the annual incidence of CM is estimated at 100,000 persons [2]. Mortality from meningitis in SSA is estimated at 19–68% with 75% of cases due to CM [3]. In Uganda alone, the annual incidence of CM is estimated at 5553 cases, assuming an 8.6% risk among PLHIV [4].

The diagnosis of CM requires a high index of clinical suspicion especially among at-risk populations and in areas with a known high burden of the disease [5]. Conventional methods for diagnosing CM include lumbar puncture (LP) with measurement of cerebrospinal fluid (CSF) opening pressure, glucose, protein, cell counts, India ink staining, microscopy, culture of CSF pellet after centrifugation which is the gold standard and brain imaging [6]. The conventional methods are quite slow and can delay diagnosis and hence treatment of patients which eventually affects the prognosis [7]. Over time, the laboratory methods for CM diagnosis have evolved from the use of the conventional methods to the use of rapid diagnostics that detect cryptococcal antigen (CrAg) in CSF [8].

These rapid assays are affordable, require no technical expertise and provide results within 10 min [9]. They are additionally suitable as point-of-care tests and trained personnel can perform the tests in a health facility or at the patient’s bedside [10]. The current algorithm for diagnosing CM incorporates these rapid assays, fundamental in early diagnosis and effective management of CM [11]. Several laboratory tests that detect the CrAg are commercially available, with the IMMY CrAg LFA being the most widely used [10].

Among PLHIV, the IMMY assay has a sensitivity of 98.8% in CSF, superior to both India ink and culture [6]. The Dynamiker CrAg LFA, a rapid assay for diagnosis of CM has an estimated sensitivity of 100%, specificity of 89.9% and 90.7% diagnostic accuracy [12]. A comparative study estimated the specificity of IMMY CrAg LFA at 98.3% versus 97% specificity with the Dynamiker CrAg LFA [13]. IMMY is not the gold standard and thus not the ultimate comparative assay for validating Dynamiker. A few studies in Africa, and none so far in Uganda, have validated Dynamiker with reference to the gold standard. Therefore, this study aimed at evaluating the diagnostic performance, time to results and average cost of Dynamiker CrAg LFA in screening for CM, using CSF culture as the reference standard, in a high-burdened CM Ugandan setting.

## Methods

### Study design

This was a cross-sectional study nested within an ongoing observational cohort at the Infectious Diseases Institute (IDI). The parent study, Improving Diagnostics and Neurocognitive Outcomes in HIV/AIDS-related Meningitis (COAST) focuses on providing an enhanced, standardized package of diagnostics for HIV-related meningitis, especially for cryptococcus, *Mycobacterium tuberculosis*, bacterial and viral pathogens, including cytomegalovirus and measuring the long-term outcomes of HIV-associated central nervous system infections in adults via annual visits. Between January and March 2025 inclusive, we tested stored CSF samples from patients enrolled under the COAST study at the IDI.

#### Sampling technique

A purposive sampling technique was employed. From the available archived specimens, samples were intentionally selected to ensure inclusion of both cryptococcal-positive and cryptococcal-negative cases. This approach was used to obtain a representative spectrum of outcomes required for evaluating the diagnostic performance of the Dynamiker CrAg LFA (Dynamiker Biotechnology, Tianjin, China), including sensitivity, specificity, predictive values and level of agreement with the reference standard.

#### Study population, sample size, inclusion and exclusion criteria

The sample size was determined by the Kish–Leslie formula using an estimated prevalence of 20% (3) at a confidence interval of 95%, obtaining a sample size of 245.

#### Study population

The study population consisted of participants enrolled under the ongoing COAST study at the IDI.

#### Inclusion criteria

We included stored CSF samples that were obtained on the screening visit for the period between January 2024 and March 2025 inclusive.

CSF samples clearly labelled with visit code 0.0 or 0.1, study ID, age, sex and date of collection were particularly included in this study.

#### Exclusion criteria

We excluded CSF samples with an insufficient volume required to run both the Dynamiker and perform CSF culture, while retaining some CSF for future studies. Insufficient volume CSF samples excluded were those less than 500 µl.

### Laboratory methods

Procedures for Dynamiker CrAg LFA and CSF culture were carried out in a biosafety level-2 cabinet within the IDI’s translational laboratory and the CAP-accredited mycology laboratory at the Makerere College of Health Sciences. Procedural details have been summarized in File S2, available in the online Supplementary Material.

### Procedure for Dynamiker CrAg LFA

A Dynamiker test kit was obtained and placed at room temperature on a clean flat surface within a biosafety cabinet. The kit was labelled with a sample ID, corresponding to that of the thawed, vortexed CSF samples. Using a sterile dropper packaged together with the kit, three drops of CSF were added into the sample pad of the Dynamiker kit and the test was left to run for 15 min and not more than 20 min. Positive and negative controls were run using the standard positive and negative control solutions that came packaged per box of 50 kits of the Dynamiker test kits. Results were read and recorded as positive if two red lines, the test line (T) and the control line (C) were present; negative if a single control line (C) and no red test line appeared; and invalid if no control line (C) was observed.

### Procedure for CSF culture

Plates of Sabouraud’s dextrose agar (SDA) (Condalab, Madrid, Spain) were transferred to the working area in a biosafety cabinet. These were clearly labelled with the laboratory number and date of inoculation. The remainder of the CSF was centrifuged at 3,000 r.p.m for 10 min. The supernatant was carefully decanted into a clean vial, taking extra care not to dislodge the deposit. Using a sterile loop, the deposit was inoculated onto the labelled SDA. A parafilm tape was applied to the inoculated plate to minimize possible contamination of the plate by moulds and bacteria. The plate was then incubated at 37 °C aerobically and the time of incubation was recorded. The plates were monitored for growth at 24, 48 and 72 h and every 24 h for up to 14 days if no growth at 72 h. Absence of growth by day 14 indicated negative culture results. For positive cultures, evidenced by the appearance of opaque, smooth, mucoid, white to cream colonies, further identification tests were performed, including India ink (Becton Dickinson, New Jersey, USA) staining and the urease test to confirm the identity of the organism.

### Procedure for India ink staining

India ink staining was performed to confirm the identity of the organism isolated on SDA. A drop of India ink stain was placed onto a clean microscope slide. Using a sterile loop, a colony was obtained and transferred onto the drop of India ink stain on the slide and mixed carefully, taking care not to form bubbles. A coverslip was then applied and the slide examined under X10 for encapsulated yeast cells.

### Procedure for urease test

The urease test was carried out to further confirm the presence of *Cryptococcus* spp*.* capable of producing urease which hydrolyses the urea in the medium to produce ammonia. The ammonia was responsible for the colour change in the medium that indicated a positive result. Urease agar was inoculated with a colony of the organism using a sterile loop and incubated at 37 °C for 24–72 h, examining for changes in the agar every 24 h. Results were recorded as urease positive for changes from the yellow colour of the medium to pink and negative for no changes in colour.

### Quality control

For Dynamiker CrAg LFA, internal procedural controls included in the test were used. A coloured band appearing in the control region (C) was considered as an internal positive procedural control. It confirmed sufficient specimen volume and correct procedural technique. Positive and negative controls were performed for culture by inoculation of SDA with known strains of Cryptococcus and sterile saline, respectively.

### Data management

Data were recorded into Microsoft Excel (File S1), where it was checked for correctness and completeness before exporting it to the different software for analysis.

### Data analysis

Data were analysed using STATA version 17 (STATA, College Station, TX). Statistical analysis was aimed at comparing the sensitivity, specificity, positive predictive value, negative predictive value, level of agreement and kappa test for reproducibility in the results of the Dynamiker CrAg LFA compared to CSF culture as the reference standard. The average time to results was computed in Microsoft Excel Office 2011. Baseline clinical and demographic data for participants were summarized as median (interquartile range, IQR) for continuous variables and n (%) for categorical variables.

## Results

### Study population characteristics

Two hundred and forty-five CSF samples from 200 PLHIV were included in this study. All participants had advanced HIV disease with a median CD4 + T cell count of 34 cells/µl (IQR=12–112). The median age of participants was 37 years (IQR=30–43). 46% (91/200) of the participants were female and the majority (65%) were antiretroviral therapy (ART) naïve (Table 1).

**Table 1.** Baseline characteristics for study participants

Characteristic	N	Statistic
**Age (years), median (IQR**)	200	37 (30, 43)
**Gender (female), n (%)** **(male), n (%)**	200	91 (46%) 109 (54%)
**Weight (kg), median (IQR)**	198	55 (50, 61)
**GCS<15, n (%)**	198	99 (50%)
**ART group, n (%)** **Currently on ART** **Never on ART** **Previously on ART**	186	47 (25%) 121 (65%) 18 (9.7%)
**Weeks on ART, median (IQR)**	47	8 (2,140)
**Headache, n (%)**	200	169 (85%)
**Nuchal rigidity, n (%)**	199	95 (48%)
**Mania, n (%)**	200	12 (6%)
**Confusion, n (%)**	200	128 (64%)
**Photophobia, n (%)**	200	47 (24%)
**Focal neurological deficits, n (%)**	199	34 (17%)
**Visual changes, n (%)**	200	70 (35%)
**Seizures, n (%)**	200	53 (27%)
**CD4 + count (cells/µl), median (IQR)**	158	34 (12, 112)
**Haemoglobin (g/dl), median (IQR)**	172	11.30 (9.70, 13.20)
**Creatinine (mg/dl), median (IQR)**	168	0.72 (0.58, 0.94)
**Sodium (mmol/l), median (IQR)**	172	133 (129, 137)
**Potassium (mmol/l), median (IQR)**	172	4.10 (3.60, 4.50)
**Sterile at admission, n (%)**	193	97 (50%)
**CSF white blood cell (WBC) count (cells/µl), median (IQR)**	192	33 (4, 128)
**CSF protein (mg/dl), median (IQR)**	191	80 (40, 115)
**CSF glucose (mg/dl), median (IQR**)	191	68 (47, 97)
**CSF lactate (mg/dl), median (IQR**)	184	3.20 (2.40, 5.00)
**Opening pressure (mmH_2_O), median (IQR**)	175	200 (115, 315)
**Closing pressure (mmH_2_O), median (IQR**)	159	70 (50, 100)

Data are presented as n (%); median (interquartile range).

ART, Antiretroviral therapy; CD4+, Cluster of differentiation 4; CSF, cerebrospinal fluid; GCS, Glasgow Coma Scale; IQR, Interquartile range.

### Culture results (reference standard)

Of the 245 CSF samples, 105 (42.9%) yielded *Cryptococcus* species growth upon fungal culture, while 140 (57.1%) were culture negative (Table 2).

**Table 2.** A 2×2 contingency table for the evaluation of Dynamiker CrAg LFA using CSF culture as the reference standard

		CSF culture		
		Positive	Negative	Total
**Dynamiker CrAg LFA**	**Positive**	105	21	126
	**Negative**	0	119	119
	**Total**	105	140	245

CrAg, Cryptococcal antigen; CSF, Cerebrospinal fluid; LFA, Lateral flow Assay.

### Dynamiker CrAg lateral flow assay results

From the Dynamiker CrAg LFA, 126 positive and 119 negative results were obtained (Table 2).

### Diagnostic performance of Dynamiker CrAg LFA

Using fungal CSF culture as the reference standard, the diagnostic performance of Dynamiker CrAg LFA was summarized in Table 3. The assay demonstrated excellent sensitivity and negative predictive value, while the specificity and positive predictive value were slightly lower, influenced by 21 false positive results (8.6%). No false negative results were observed.

**Table 3.** Diagnostic performance of Dynamiker CrAg LFA using CSF culture as the gold standard

Variable	N	Statistic	95%** CI**	Interpretation
**Sensitivity**	245	100%	96.55–100%	Excellent
**Specificity**	245	84.89%	77.84–90.40%	Good
**PPV**	245	83.33%	77.12–88.12%	Good
**NPV**	245	100%	96.92–100%	Excellent
**Accuracy**	245	91.39%	87.14–94.59%	Excellent
**Kappa**	245	0.829	0.760–0.898%	Almost perfect agreement
**% False positives**	245	8.6%	4.3–14.5%	Good
**% False negatives**	245	0%	0–0.03%	Excellent

CI, Confidence Interval; NPV, Negative predictive value; PPV, Positive predictive value.

### Time to results

The median time to final culture result was 3 days (IQR: 2–3) with a mean of 2.33 days (sd: 0.85). The median time to results of the Dynamiker CrAg LFA was 30 s (IQR: 18–65) with a mean of 30.03 s (sd: 14.61)

### Cost of performing Dynamiker CrAg LFA compared to CSF culture

The unit cost of a Dynamiker CrAg LFA test kit is ~2 USD when procured through institutional channels in resource-limited settings [14]. Additional charges for importation, shipping and personnel time, when taken into consideration, increase the total estimated cost per test in Uganda to ∼3.41 USD [15]. In private diagnostic laboratories in Uganda, the average cost of a CSF culture and sensitivity test is approximately UGX 60,000, which is equivalent to ∼15.80 USD. Additional confirmatory tests, such as India ink preparation, further increase the diagnostic expense, costing between UGX 15,000 and UGX 25,000 (USD 4.00 to USD 6.60) (Grace Laboratories, 2024).

## Discussion

### Diagnostic performance of Dynamiker CrAg LFA compared to culture

Findings from this study showed that the Dynamiker CrAg LFA is a rapid and accurate diagnostic test for HIV-associated CM. The excellent sensitivity and good specificity when compared to the reference standard of fungal culture of CSF implied that the Dynamiker CrAg LFA is a highly reliable test in detecting true cases of CM. This makes it a potentially valuable tool for the early and rapid diagnosis of CM. The perfect sensitivity indicates that the assay correctly identified all cases with culture-confirmed cryptococcal infection. This is a critical attribute for any screening tool, especially for opportunistic infections like CM in HIV-infected individuals, where delayed diagnosis is associated with poor treatment outcomes. A test with high sensitivity ensures that no case of CM is missed, which is particularly important given the often-subtle early symptoms of the disease and its rapid progression.

The observed specificity indicates that the assay gave some false positive results (21/140 CrAg positive cases were culture negative). While not ideal, this is quite a good specificity as a majority of the individuals without the disease were correctly identified. However, false positives may result in over-treatment, unnecessary antifungal therapy and further diagnostic procedures. On the other hand, some of the culture-negative but CrAg-positive results may be a result of subclinical infection, prior antifungal therapy and low fungal burden below the culture detection threshold. The specificity of the assay may also have been affected by the use of stored samples in this study, which could have had significant effects on the viability of the yeast cells.

The positive predictive value indicated that ~5 in 6 patients who tested positive with the Dynamiker CrAg LFA truly had CM. The negative predictive value emphasizes the reliability of this rapid test in ruling out CM. The level of agreement observed between the Dynamiker CrAg LFA and culture proved a very good agreement beyond chance. Overall, the test had a good diagnostic accuracy when compared to fungal CSF culture.

The diagnostic performance of the Dynamiker CrAg LFA based on this study was consistent with findings from other studies [12,16, 17]

### Time to results

The assay demonstrated a rapid turnaround time and was also easy to use, with no need for sophisticated equipment, making it ideal for point-of-care testing and for the prompt diagnosis of CM in resource-limited settings with a significant burden of the disease.

### Cost of performing dynamiker compared to culture

The Dynamiker CrAg LFA has a relatively low charge per test that makes it feasible to implement screening, even at lower-level health facilities with limited laboratory capacity. CSF culture, although considered the reference standard for confirming CM, is significantly more expensive and labour-intensive. It involves inoculation onto Sabouraud dextrose agar, incubation for at least 3 days and microscopic identification of yeast colonies. All these procedures require a functional microbiology laboratory, trained personnel and reliable power supply, resources that may not be consistently available in many lower-level Ugandan healthcare facilities. Beyond the direct costs, culture-based methods also incur opportunity costs due to delayed turnaround time, which can impede rapid clinical decision-making and patient management. In contrast, the Dynamiker CrAg LFA can be completed within seconds, facilitating real-time diagnosis and initiation of therapy.

### Study limitations

Although culture is historically considered the gold standard, its sensitivity is largely dependent on the fungal burden. A relatively low fungal burden, such as occurs in the initial stages of infection, and in cases of prior antifungal therapy, results in negative cultures. This could have contributed to the false positive cases observed in this study. The use of stored samples in this study could also have limited the diagnostic accuracy of the test.

### Conclusions

The Dynamiker CrAg LFA demonstrated an excellent sensitivity and a good negative predictive value, making it a highly effective tool for screening for HIV-associated CM. Its ease of use with minimal technicalities and the rapid time to results make it ideal for utilization in resource-limited settings. Because the confidence intervals are wide for all parameters, the two tests perform equally well, with Dynamiker having an excellent diagnostic accuracy.

### Recommendations

We recommend that the Dynamiker CrAg LFA be considered for inclusion in cryptococcal screening programmes especially in high-prevalence settings. In cases where the Dynamiker test is positive but culture is negative, additional laboratory testing can be done to guide treatment decisions. Larger studies across diverse clinical settings are also recommended to validate this test for generalizability of findings. Future studies validating the test in reference to the historical gold standard of CSF culture should consider the use of fresh CSF samples, and should incorporate patient follow-up to tailor findings with clinical history and patient outcomes especially in culture-negative, CrAg-positive cases. More studies with larger sample sizes are recommended to facilitate a policy statement concerning the Dynamiker CrAg LFA test.

## Supplementary material

10.1099/jmm.0.002173 Supplementary Material 1.

10.1099/jmm.0.002173 Supplementary Material 2.

## Abbreviations

AIDS: acquired immunodeficiency syndrome
CD4+: cluster of differentiation 4
CM: cryptococcal meningitis
COAST: improving diagnostics and neurocognitive outcomes in HIV/AIDS-related meningitis
CrAg LFA: cryptococcal antigen lateral flow assay
CSF: cerebrospinal fluid
CSF: cerebrospinal fluid
FDA: Food and Drug Administration
GCS: Glasgow Coma Scale
HIV: human immunodeficiency virus
IDI: Infectious Diseases Institute
IQR: interquartile range
LP: lumbar puncture
NPV: Negative predictive value
PLHIV: people living with HIV
PPV: Positive predictive value
SDA: Sabouraud’s dextrose agar
SSA: Sub-Saharan Africa
